# Expression of pre-selected TMEMs with predicted ER localization as potential classifiers of ccRCC tumors

**DOI:** 10.1186/s12885-015-1530-4

**Published:** 2015-07-14

**Authors:** Tomasz Wrzesiński, Malgorzata Szelag, Wojciech A. Cieślikowski, Agnieszka Ida, Rachel Giles, Elżbieta Zodro, Joanna Szumska, Joanna Poźniak, Zbigniew Kwias, Hans A.R. Bluyssen, Joanna Wesoly

**Affiliations:** 1 Laboratory of High Throughput Technologies, Institute of Molecular Biology and Biotechnology, Faculty of Biology, Adam Mickiewicz University, Umultowska 89, 61-614 Poznan, Poland; 2 Department of Human Molecular Genetics, Institute of Molecular Biology and Biotechnology, Faculty of Biology, Adam Mickiewicz University, Umultowska 89, 61-614 Poznan, Poland; 3 Department of Urology and Urological Oncology, Poznan University of Medical Sciences, Szwajcarska 3, 61-285 Poznan, Poland; 4 Department of Nephrology and Hypertension, University Medical Center, Postbus 85500, 3508 GA Utrecht, Netherlands

**Keywords:** ccRCC, Transmembrane proteins, ER, Gene expression, Bioinformatics

## Abstract

**Background:**

*VHL* inactivation is the most established molecular characteristic of clear cell renal cell carcinoma (ccRCC), with only a few additional genes implicated in development of this kidney tumor. In recently published ccRCC gene expression meta-analysis study we identified a number of deregulated genes with limited information available concerning their biological role, represented by gene transcripts belonging to transmembrane proteins family (TMEMs). TMEMs are predicted to be components of cellular membranes, such as mitochondrial membranes, ER, lysosomes and Golgi apparatus. Interestingly, the function of majority of TMEMs remains unclear. Here, we analyzed expression of ten TMEM genes in the context of ccRCC progression and development, and characterized these proteins bioinformatically.

**Methods:**

The expression of ten TMEMs (*RTP3*, *SLC35G2*, *TMEM30B*, *TMEM45A*, *TMEM45B*, *TMEM61*, *TMEM72*, *TMEM116*, *TMEM207* and *TMEM213*) was measured by qPCR. *T*-test, Pearson correlation, univariate and multivariate logistic and Cox regression were used in statistical analysis. The topology of studied proteins was predicted with Metaserver, together with PSORTII, Pfam and Localizome tools.

**Results:**

We observed significant deregulation of expression of 10 analyzed TMEMs in ccRCC tumors. Cluster analysis of expression data suggested the down-regulation of all tested TMEMs to be a descriptor of the most advanced tumors. Logistic and Cox regression potentially linked TMEM expression to clinical parameters such as: metastasis, Fuhrman grade and overall survival. Topology predictions classified majority of analyzed TMEMs as type 3 and type 1 transmembrane proteins, with predicted localization mainly in ER.

**Conclusions:**

The massive down-regulation of expression of TMEM family members suggests their importance in the pathogenesis of ccRCC and the bioinformatic analysis of TMEM topology implies a significant involvement of ER proteins in ccRCC pathology.

**Electronic supplementary material:**

The online version of this article (doi:10.1186/s12885-015-1530-4) contains supplementary material, which is available to authorized users.

## Background

Renal cell carcinoma (RCC) is a relatively common malignancy accounting for approximately 2 % of all adult cancers and causing approximately 100,000 deaths per year worldwide [1]. 80 % of renal cell carcinoma (RCC) cases are classified as clear cell renal cell carcinoma (ccRCC), originated from proximal convoluted tubule, with annually 168,000 newly diagnosed cases [2]. During last 30 years the incidence of RCC has been steadily increasing, likely due to the higher exposition to risk factors such as cigarette smoking, obesity, hypertension and accidental diagnosis due to improved visualization techniques [3].

Familial, associated with von Hippel-Lindau (VHL) syndrome, and sporadic ccRCC have been linked to inactivation of *VHL* gene by mutation, loss of heterozygosity (LOH) and promoter hypermethylation [4–6]. *VHL* inactivation is the most established cause of ccRCC, but there are several other factors involved in pathogenesis of tumor type exemplified by mutations in *KNG1* and *MT1A* genes [7], as well as PI3K/AKT/mTOR signaling pathway factors (*MTOR*, *PTEN*, *PIK3CA)* [8].

One group of ccRCC deregulated genes contains gene transcripts belonging to TMEM (transmembrane proteins) gene family. Differential regulation of TMEMs could be observed in many cancers, such as lymphomas (*TMEM176*) [9], colorectal cancer (*TMEM25*) [10], meningiomas (*TMEM30B*) [11], paragangliomas and pheochromocytomas (*TMEM127*) [12]. In case of ccRCC, up- or down-regulation of different TMEMs is supported by a number of microarray experiments [13–19].

In our recently published microarray-based meta-analysis, we identified a number of up- and down-regulated genes with limited information available concerning their biological role. We found 10 significantly deregulated genes (8 down- and 2 up-regulated) which were represented by gene transcripts belonging to TMEM gene family, with highest down-regulation of *TMEM213* (effect size = −11.7) and highest up-regulation of *TMEM45A* (effect size = 2.6) [20]. TMEMs are a group of ca. 310 different proteins (with ca. 440 identified isoforms and ca. 580 transcript variants) predicted to be components of cellular membranes, such as mitochondrial membranes, lysosomes and Golgi apparatus. Interestingly, the function of majority of TMEM proteins remains unclear, mainly due to difficulties in extraction and purification of transmembrane proteins. Nevertheless many TMEM proteins have been functionally assigned as trans-membranous anion channels (e.g., ANO1) [21] and molecules responsible for oncosis (TMEM123) [22], protein glycosylation (TMEM165) [23], pathogen intoxication (TMEM181) [24], as well as innate immunity response (TMEM173) [25]. Although down-regulation of TMEMs has been detected in large number of microarrays on ccRCC tumors, not much is known about their function in pathogenesis of ccRCC.

Research performed by Kholodnyuk *et al.* showed that *RTP3* (*TMEM7*) expression in 5 different RCC cell lines - KH39, CAKI-1, CAKI-2, KRC/Y and TK-10 was impaired in comparison to normal human kidney by RT-PCR [26] and was suggested to act as of tumor suppressor gene. Additionally, overexpression of *SLC35G2* (*TMEM22*) and *RAB37* genes in ccRCC tumors was observed [27, 28] and experiments on RCC cell lines implied that the SLC35G2/RAB37 complex was likely to play a crucial role in growth of RCC [29].

Here we compared expression of 10 TMEM family members: *RTP3*, *SLC35G2*, *TMEM30B*, *TMEM45A*, *TMEM45B*, *TMEM61*, *TMEM72*, *TMEM116*, *TMEM207* and *TMEM213* in tumors histopathologically classified as ccRCC. Additionally, we correlated expression of these genes with *VHL*, *HIF1A* and *EPAS1* expression. We also analyzed expression of 10 TMEM genes in PBMCs of patients with metastatic and non-metastatic ccRCC, at the time of nephrectomy and a year post-nephrectomy. Moreover, we tested if expression of TMEM could be utilized as a potential predictor for metastases, progression-free disease course and patient survival rate. Lastly, we tried to predict topology and tertiary structure of TMEMs using bioinformatic tools.

## Methods

### Patient material collection

The samples were obtained prospectively from 76 histopathologically confirmed ccRCC tumors (Additional file 1: Table S2). Collected tissue represented cross-section of kidney tissue (i.e., inner tumor mass, border of a tumor and non-tumoral kidney tissue). Analyzed tissue represented inner but not necrotic tumor tissue. Tissues were suspended in RNALater® reagent (Sigma-Aldrich, St. Louis, MO, USA). Peripheral blood from ccRCC patients was collected before nephrectomy and ca. a year post surgery (Additional file 2: Table S3). Mean time of a follow-up was equal to 13.31 months, with median 12.5 months and range 3–34 months. Tissues and PBMCs (Peripheral Blood Mononuclear Cells) samples were obtained from the Department of Urology and Urological Oncology, Poznan University of Medical Sciences. This research was approved by Local Bioethical Committee at Poznan University of Medical Sciences (Poland), no. 1124/12 and written informed consent to participate in the study and to publish individual clinical data (including age and gender) was obtained from all patients. Disease progression was defined as local neoplasm recurrence or distant metastasis detected by at least one of following methods: X-ray, abdominal ultrasound and computer tomography of chest and abdomen. Detailed patient clinical characteristics are presented in Table 1.

**Table 1:** Characterization of ccRCC patient cohort. Median age of healthy donors which PBMCs were obtained from was 42.50 years old, 25 % Percentile = 35, 75 % Percentile = 46.75. Abbreviations: NM – not measured

Name of the characteristic	Amount		
	Tumor tissue	PBMCs before nephrectomy	PBMCs 12 months post-nephrectomy
Number of samples included in the study			
Patients	76	66	27
Controls	23	14	14
Gender			
Males (%)	45 (59)	45 (68)	19 (70)
Females (%)	31 (41)	21 (32)	8 (30)
Age (at a time of surgery)			
Median	64	65	61
25 % Percentile	57	56	53
75 % Percentile	72	73	69
Tumor size [mm]			
Median	43	43.50	44
25 % Percentile	33	31.88	28.50
75 % Percentile	65.38	65	59
TNM stage – pT			
pT1 (%)	40 (52)	34 (51)	16 (59)
pT2 (%)	2 (3)	3 (5)	2 (8)
pT3 (%)	32 (42)	28 (42)	9 (33)
pT4 (%)	2 (3)	1 (2)	0 (0)
TNM stage – pN			
pN0 (%)	73 (96)	63 (95)	27 (100)
pN1 (%)	3 (4)	3 (5)	0
TNM stage – M			
M0 (%)	62 (82)	52 (79)	25 (93)
M1 (%)	14 (18)	14 (21)	2 (7)
Fuhrman grade			
G1 (%)	4 (6)	4 (6)	0 (0)
G2 (%)	35 (46)	32 (48)	17 (63)
G3 (%)	26 (34)	22 (33)	9 (33)
G4 (%)	11 (14)	8 (13)	1 (4)
VHL expression			
VHL+	33	NM	NM
VHL-	43	NM	NM

### RNA isolation from tissues and PBMCs

Tissue total RNA was isolated from homogenized tumor tissue using GeneMATRIX Universal RNA Purification Kit (EurX, Gdańsk, Poland) following supplied protocol. PBMC RNA was isolated using Ficoll gradient (GE Healthcare, Little Chalfont, Buckinghamshire, England) followed by RNA extraction using TRI Reagent® (Molecular Research Center, Cincinnati, OH, USA). RNA quality and quantity was determined with NanoDrop Spectrophotometer ND-1000 (Thermo Scientific, Wilmington, DE, USA).

### Reverse transcription and real-time PCR

1 μg of RNA was reversely transcribed using RevertAid™ First Strand cDNA Synthesis Kit with Random Hexamers (Thermo Scientific Fermentas, Waltham, MA, USA) following supplied protocol.

Analyses were performed using Eco Real Time System (Illumina, San Diego, CA, USA), with Maxima™ SYBR Green/ROX qPCR Master Mix (2×) (Thermo Scientific Fermentas, Waltham, MA, USA) following supplied protocol. Primers were designed using Primer-BLAST (http://www.ncbi.nlm.nih.gov/tools/primer-blast) and Oligo Analyzer 3.1 (http://eu.idtdna.com/analyzer/applications/oligoanalyzer/default.aspx). Analyzed samples were corrected by reaction efficiency obtained from standard curves which varied from 91 % to 110 %. Primers used for the analyses are listed in Additional file 3: Table S1. Expression of each gene in ccRCC tissue samples and PBMCs was measured in duplicates, in two independent experiments. Calculated normalized relative quantities (CNRQ) of transcripts were obtained by normalization to reference gene (*ACTB*) and average of reference samples using qBase Plus software (https://www.biogazelle.com/qbaseplus).

### Statistical analysis

All charts were prepared using GraphPad Prism software v6.02 (http://www.graphpad.com). IBM SPSS Statistics software v22 (http://www-01.ibm.com/software/analytics/spss/products/statistics/) was used for *t*-test, ANOVA and Pearson correlations. For clusters analysis TMEM expression values were dummy-coded basing on their deviation from average expression among all tumor samples. Univariate and multivariate logistic regression, and univariate and multivariate Cox regression were performed using R statistical software v3.1.1 [30]. For regression models, AIC (Akaike information criterion) [31] was used to assess the relative quality of each predictor, as compared to the null model. Firstly, in case of multivariate logistic regression, maximum 4 best predictors was chosen (to prevent a model from overfitting) based on best subset selection using *leaps* package [32] and the lasso shrinkage method using *glmnet* package [33, 34]. Each variable were tested for colinearity using variance inflation factor (VIF) calculation [35]. Then, each coefficient was recalculated using penalized maximum likelihood estimation to avoid overfitting of a model by *rms* package [36–40]. To assess true standard error and prediction capabilities of each model bootstrap and cross-validation methods were used, as implemented in *rms* library [37]. Univariate Cox regression to assess the marginal effect of each factor (when not correcting for the effect of other factors) were performed. Multivariate Cox regression was calculated by *rms* package and variables in the model were included based on step-wise method and the lasso shrinkage method using *glmnet* package [41]. Each variable included in Cox regression model was tested for proportional hazards assumption based on Grambsch and Therneau method [42]. All p-values obtained during statistical analyses (i.e., from t-tests, ANOVA, Pearson correlations, univariate and multivariate logistic and Cox regression models) were adjusted all together for multiple comparisons using Benjamini-Hochberg method [43], and obtained q-values were considered to be statistically significant at q ≤ 0.1.

### Bioinformatic prediction of TMEMs topology and function

*Query sequences selection*: The following NCBI RefSeq sequences of native human TMEMs were used for bioinformatics analysis, NCBI GIs: 146229352 (TMEM213), 46409276 (TMEM207), 32698902 (TMEM61), 20270331 (TMEM45B), 63003930 (TMEM30B), 13899263 (RTP3), 183227675 (TMEM72), 302058299 (TMEM116), 148224156 (SLC35G2) and 8922243 (TMEM45A). *Pfam analysis*: Query sequences were submitted to the Pfam 27.0 database of protein families and Hidden Markov model HMM searches (default Pfam-A data set and e-value of 1.0) were conducted [44]. *Fold recognition analysis Metaserver*: Full-length sequences were submitted to Genesilico Metaserver gateway [45]. The consensus of different programs and servers was used to determine transmembrane helices, protein order and secondary structure, using 12, 16 and 17 methods, respectively. Protein solvation prediction was determined with 6 different programs and relative solvent accessibility of amino acid (RSA, %). An aa was considered buried with RSA value lower than a assumed, implemented threshold (25 %, 5 %, or 0 %). If RSA value for the aa was higher than the threshold, it was considered as exposed. TMEM homologs with tertiary structures deposited in RCSB PDB were determined by fold-recognition methods, which were compared, evaluated, and ranked by the Pcons5 algorithm [46]. *PSORT II:* Sequences were submitted and analyzed based on their amino acid sequence [47]. The presence of signal sequences was determined by recognizing positively charged N-region, the central hydrophobic region and searching the position of possible cleavage site [48, 49]. TM segments were predicted with ALOM method and the membrane topology was scored using Singer’s classification [50, 51]. The cytoplasmic/nuclear discrimination for TMEMs was analyzed by Reinhardt’s algorithm, which determines the probability of protein localization by `NNCN score` [52]. *Localization assignment*: mitochondrial [47], nuclear [52], peroxisomal [53], endoplasmic reticulum [54], lysosomal and vacuolar [55] were presented as consensus with the use of k-nearest neighbor (k-NN) algorithm [56]. *Localizome*: TM helix number and topology was predicted with Phobius algorithm [57, 58]. The hmmpfam method was employed (default e-value of 0.01) to include domains obtained from Pfam. Combined results were validated by LocaloDom database of protein domains. *Consensus analysis*: Metaserver, PSORT II and Localizome predictions of TMEMs were superimposed. Obtained colocalization of the data was analyzed to find the most probable protein topology and evaluate the reliability of the calculations.

## Results

### TMEM expression in tumor tissue

**Fig. 1:**
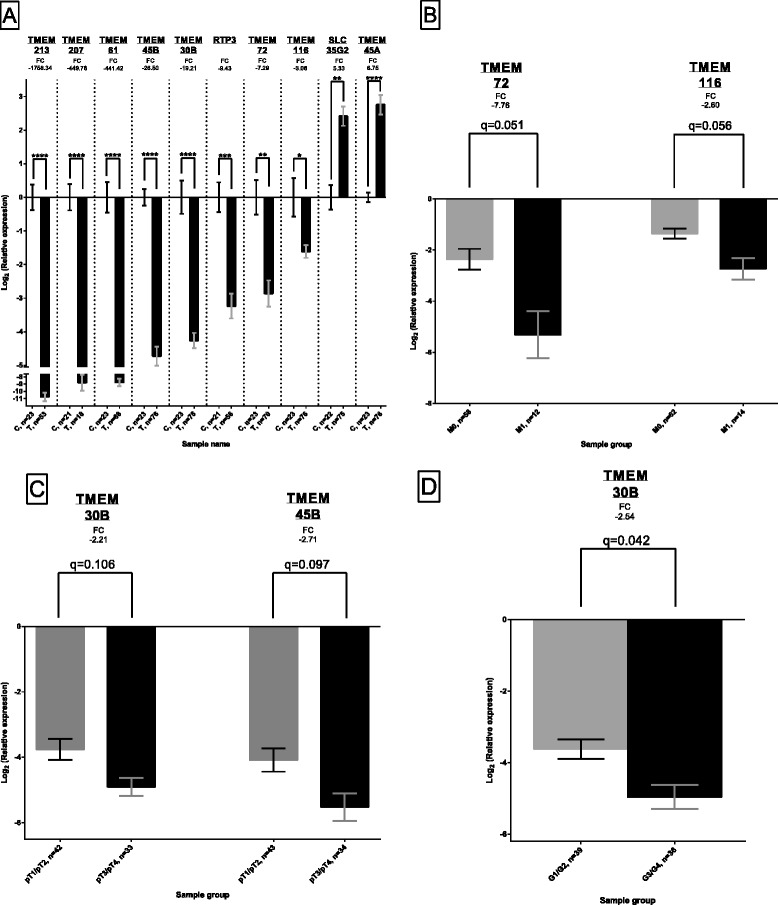
Differential expression of TMEMs in tumor tissue. (**a**) A comparison of TMEM expression between a tumor tissue and healthy kidney tissue. (**b**) TMEM expression in metastatic and non-metastatic tumors. (**c**) TMEM expression in organ-confined and advanced stage tumors (**d**) TMEM expression in low Fuhrman grade and high Fuhrman grade samples. Average log_2_ relative expression data in each sample group ± standard error of mean is shown in each chart. FC – fold-change. n – number of samples. T – tumor tissue samples. C – healthy tissue samples. M0 – non-metastatic ccRCC. M1 – metastatic ccRCC tissues. pT1/pT2 – organ-confined tumors, as assessed by TNM staging system. pT3/pT4 – advanced tumors, as assessed by TNM staging system. G1/G2 – low Fuhrman grade samples. G3/G4 – high Furhman grade samples. * - q < 0.05. ** - q < 0.01. *** - q < 0.001. **** - q < 0.0001

In line with our previous observations, we detected statistically significant differences in expression of all ten TMEMs in tumor tissue and healthy kidney tissue (Fig. 1a) [20]. The down-regulation varied between the genes, with 1758.34 fold for *TMEM213* (q < 0.0001) and 3.06 fold for *TMEM116* (q < 0.05). Both, *SLC35G2* and *TMEM45A* were found to be up-regulated with 5.33 (q < 0.01) and 6.75 fold change (q < 0.0001), respectively. We investigated if analyzed TMEMs displayed differential expression in metastatic and non-metastatic tumors. As shown in Fig. 1b, we detected significant difference in expression of *TMEM72* and *TMEM116*: 39.78 fold and 6.65 fold down-regulation in metastatic tissue (*n* = 12 and *n* = 14, respectively) as compared to 5.13 fold and 2.57 fold in the non-metastatic samples (*n* = 58, q = 0.051 and *n* = 62, q = 0.056, respectively). Next, we subdivided samples into organ-confined tumors (pT ≤ 2) and advanced tumors (pT ≥ 3). A decrease of *TMEM30B* (q = 0.106) and *TMEM45B* (q = 0.097) expression was found in advanced-stage samples, with −2.21 and −2.71 fold-change, as compared to early-stage tumors, respectively (Fig. 1c). T-test was used to compare TMEM expression in well differentiated tumors (Fuhrman grade, G ≤ 2) with undifferentiated tissues (G ≥ 3, Fig. 1d) and we observed a stronger down-regulation of *TMEM30B* in high Fuhrman grade samples (fold-change −31.18, *n* = 36) in contrast to low Fuhrman grade samples (fold-change −12.29, *n* = 39, q < 0.05). Expression comparisons of all analyzed 10 TMEM genes in metastatic and non-metastatic tumors, early- and late stage samples, and tumors with undifferentiated and well-differentiated tissues are shown in Additional file 4, 5 and 6: Figures S1-S3, respectively.

**Fig. 2:**
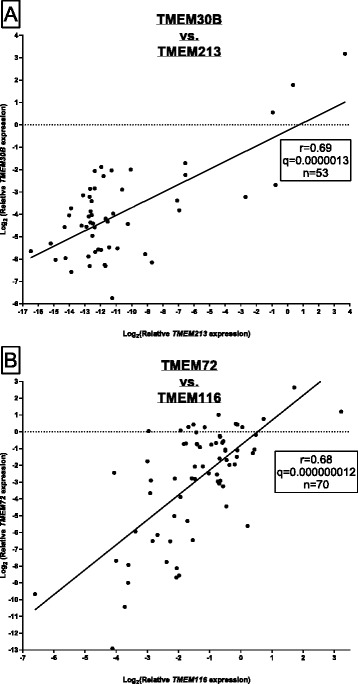
Correlations for the expression of TMEM genes in tumor tissue. (**a**) Pearson correlation between the expression of *TMEM30B* and *TMEM213*. (**b**) Pearson correlation between the expression of *TMEM72* and *TMEM116*. r – Pearson correlation coefficient. q – q-value, n – number of samples

Pearson correlation of TMEMs and *VHL*, *HIF1A* and *EPAS1* expression values was performed but correlation coefficient did not exceed 0.55 (in case of *EPAS1* expression correlated with *SLC35G2* (r = 0.55), *TMEM30B* (r = 0.32), *TMEM72* (r = 0.44) and *TMEM116* (r = 0.35), data not shown). Finally, we correlated TMEM expression between each other and overall we found the highest statistically significant Pearson correlation coefficient in case of *TMEM213* with *TMEM30B* (Fig. 2a) and for *TMEM72* correlated with *TMEM116* (Fig. 2b).

**Fig. 3:**
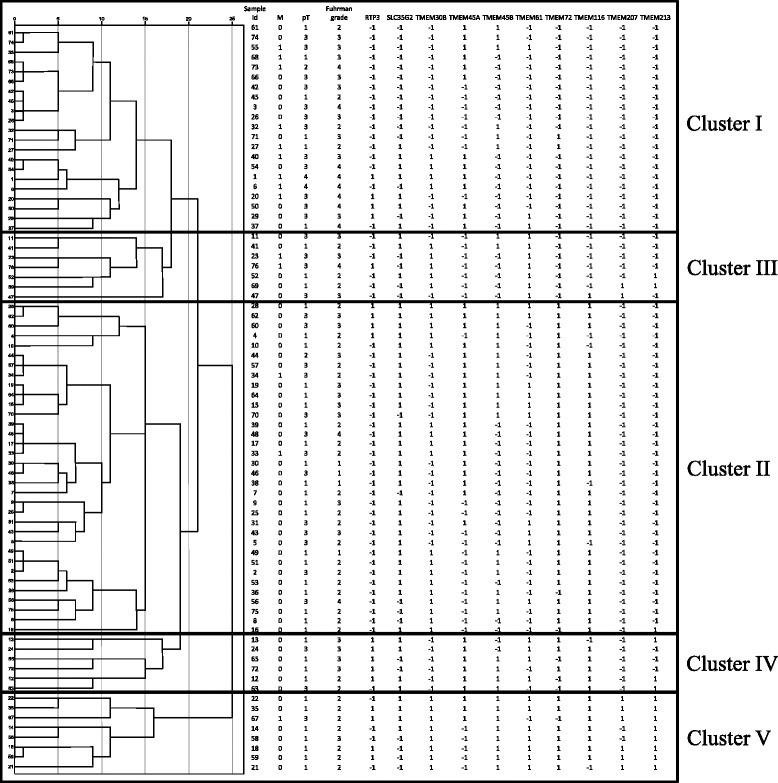
A dendrogram of hierarchical clustering of tumor samples based on TMEMs expression with denoted clusters. Clinical characteristics of samples: M – distant metastases status (0 – no distant metastases; 1 – distant metastases at the time of nephrectomy). pT – tumor size (TNM scale). Expression status of TMEM genes depicted as below average (−1) and above average (1)

In attempt to stratify the ccRCC tumors and to establish common expression profile based on TMEM expression we performed hierarchical clustering of available data (Fig. 3). TMEM expression values were dummy-coded basing on their deviation from average expression among all tumor samples as samples with TMEM expression above average (coded as “+1”) and below average (coded as “-1”). Using IBM SPSS Statistics v22 software we identified five distinctive tumor clusters. Cluster I was characterized by low expression of *TMEM72*, *TMEM116*, *TMEM207* and *TMEM213*. Samples with low expression of *RTP3*, *TMEM207* and *TMEM213* and with high expression of *SLC35G2*, *TMEM72* and *TMEM116* were preferentially assigned to cluster II. In cluster III low expression of *TMEM45A* and *TMEM72*, and high expression of *TMEM61* were observed. Low expression of *TMEM30B*, *TMEM207* and high expression of *RTP3*, *TMEM45A* and *TMEM116* were distinctive for cluster IV. Cluster V was characterized by high expression of *TMEM30B*, *TMEM45B* and *TMEM213*. Interestingly, cluster I was overrepresented by samples with more advanced disease stage (*n* = 21), as measured by tumor stage (TNM scale), Fuhrman grade and metastasis and cluster V contained less advanced tumors.

### TMEM expression in tumor tissue as independent predictors of metastases, Fuhrman grade and disease progression

We checked TMEM expression in subgroups divided according to clinical parameters (i.e., gender, smoking status, hypertension etc.) and analyzed TMEM correlation with continuous variables, such as age, average tumor size etc., but we did not find any significant differences (data not shown).

To evaluate the independent prediction of TMEM expression for clinical parameters univariate and multivariate logistic regression analyses were implemented (Table 2).

**Table 2:** Univariate and multivariate logistic regression analysis with clinical parameters and profiles of TMEM expression in tumors. (A) metastatic and non-metastatic samples. (B) Low and high Fuhrman grade samples. Confidence intervals and p-values in multivariate logistic regression were calculated using 10000-times bootstrap and were reported for penalized model. ROC AUC and R^2^ parameters from both bootstrap and cross-validation were reported. *Abbreviations:* OR – odds ratio. 95 % CI OR – 95 % confidence interval. AIC – Akaike Information Criterion. n – number of samples. ROC AUC – receiver operating characteristics area under curve parameter. VIF – variance inflation factor

(A) Metastases	Univariate							Multivariate											
	*p*-value	q-value	OR	95%CI OR		AIC	*n*	*p*-value	q-value	OR	95%CI OR		AIC	*n*	VIF	ROC AUC	R^2^	ROC AUC	R^2^
				Lower	Upper						Lower	Upper				Bootstrap		Cross-validation	
Average tumor size [mm]	0.0001	0.004	1.070	1.038	1.113	53.46	76	0.0002	0.006	1.087	1.051	1.162	38.09	70	1.195	0.919	0.565	0.932	0.800
*TMEM72* expression	0.009	0.089	0.776	0.634	0.931	60.67	70	0.0113	0.105	0.738	0.555	0.884			1.195				
Symptoms	0.002	0.032	7.500	2.199	28.511	66.19	76	Variables not in the multivariate model											
*TMEM116* expression	0.009	0.094	0.601	0.394	0.863	68.84	76												
*TMEM61* expression	0.019	0.139	0.790	0.634	0.945	60.44	68												
*EPAS1* expression	0.102	0.366	0.684	0.428	1.079	73.93	76												
(B) Fuhrman grade	Univariate							Multivariate											
	*p*-value	q-value	OR	95%CI OR		AIC	*n*	p-value	q-value	OR	95%CI OR		AIC	*n*	VIF	ROC AUC	R^2^	ROC AUC	R^2^
				Lower	Upper						Lower	Upper				Bootstrap		Cross-validation	
*EPAS1* expression	0.00004	0.002	0.235	0.107	0.433	78.70	76	0.0003	0.008	0.265	0.117	0.476	62.80	75	1.143	0.898	0.600	0.919	0.798
Smoking	0.00177	0.018	4.764	1.837	13.153	98.75	76	0.0772	0.317	3.092	0.954	12.616			1.069				
Average tumor size [mm]	0.00299	0.032	1.039	1.015	1.067	98.11	76	0.004	0.054	1.048	1.023	1.099			1.241				
*TMEM30B* expression	0.00574	0.044	0.645	0.460	0.858	97.98	75	0.02	0.146	0.634	0.351	0.825			1.286				
*VHL* expression	0.0208	0.066	0.529	0.295	0.882	103.21	76	Variables not in the multivariate model											
*TMEM72* expression	0.0255	0.150	0.832	0.699	0.970	95.22	70												
*TMEM45B* expression	0.028	0.167	0.794	0.637	0.966	103.92	76												
*TMEM116* expression	0.0408	0.180	0.725	0.520	0.970	104.58	76												
*SLC35G2* expression	0.0442	0.222	0.811	0.651	0.985	103.38	75												

To evaluate differential TMEM expression as potential predictor of distant metastases univariate logistic regression was performed. It revealed that, together with average tumor size, presence of symptoms, and expression of *EPAS1*, 2-fold increase in the expression of *TMEM72* and *TMEM116* decreased the odds of metastases significantly for 22 % and 40 %, respectively (Table 2A). Furthermore, AIC of each TMEM predictor in univariate logistic regression was lower (60.67 and 68.84 for *TMEM72* and *TMEM116* expression respectively) than AIC of null model (AIC = 74.61) suggesting potential predicting ability of analyzed variables. Removal of each of parameters (i.e., average tumor size in mm and *TMEM72* relative expression) from multivariate model lowered the prediction ability of the model, as measured by likelihood ratio test (q < 0.1). There were no collinear variables which could negatively influence prediction ability of the model, as assessed by VIF. The model was of good predictive ability as measured by ROC AUC values and R^2^ (ROC AUC = 0.919 and 0.932, R^2^ = 0.565 and 0.800, measured by bootstrap and cross-validation methods correspondingly).

*TMEM30B* expression could be associated with significant decrease in odds of having high Fuhrman grade tumor in univariate logistic regression analysis (Table 2B). It had a potential predicting ability in case of Fuhrman grade classification, as measured by AIC (*TMEM30B* expression AIC = 97.98) compared to null model (AIC = 107.31). The multivariate logistic regression revealed that 2-fold up-regulation of *TMEM30B* could be associated with significant decrease in odds of having high Fuhrman grade tumor by 37 %, in line with univariate logistic regression. Although *TMEM30B* expression status did not reach statistical significance (q > 0.1), removal of each parameter from the model, would worsen the model significantly, as seen using likelihood ratio test (q < 0.1). Bootstrapped and cross-validated predictive ability of the model was very good (ROC AUC > 0.89, R^2^ ≥ 0.6).

Univariate Cox regression (Table 3) revealed that, together with other factors, 2-fold increase of *TMEM116* expression in tumor tissues is correlated with decreased risk of disease-related mortality (hazard ratio = 0.554, q = 0.086). Although hazard ratio of *TMEM72*, *SLC35G2* and *TMEM61* expression were not statistically significant, AIC for these genes were lower (AIC = 24.52, 48.63 and 32.23, respectively) than the null model (AIC = 26.35 for samples expressing *TMEM72*, AIC = 49.45 for samples expressing *SLC35G2* and AIC = 34.47 for tumors expressing *TMEM61* above detection limit). Multivariate regression model was built to assess TMEM expression as independent predictors of ccRCC-linked mortality but probably due to small sample size (total *n* = 76, cases = 7, censored = 59, no follow-up = 10) none of predictors was associated independently with this parameter (q > 0.1). Progression-free survival was also assessed by univariate Cox regression and it revealed that expression of analyzed TMEMs could not be associated with progression-free disease course (q > 0.1, data not shown).

**Table 3:** Univariate and multivariate overall survival Cox regression model with clinical parameters and profiles of TMEM expression in tumors. Total cases = 66, censored cases = 59. *Abbreviations:* HR – hazard ratio. 95 % CI HR – 95 % confidence interval. AIC – Akaike Information Criterion. n – number of samples. n events – number of deaths (i.e., cases not censored). VIF – variance inflation factor

Overall survival	Univariate							Multivariate							
	*p*-value	q-value	HR	95 % CI HR		AIC	*n* (*n* events)	*p*-value	q-value	HR	95 % CI OR		AIC	*n* (*n* events)	VIF
				Lower	Upper						Lower	Upper			
*TMEM116* expression	0.008	0.086	0.554	0.358	0.856	44.85	66 (7)	0.082	0.319	7.167	0.777	66.147	18.60	61 (4)	4.915
Average tumor size [mm]	0.009	0.089	1.044	1.011	1.077	44.41	66 (7)	0.022	0.153	1.133	1.018	1.260			2.788
*VHL* expression	0.028	0.180	0.504	0.273	0.931	47.03	66 (7)	0.223	0.549	0.379	0.079	1.807			2.430
*TMEM72* expression	0.048	0.186	0.736	0.543	0.997	24.52	61 (4)	0.045	0.239	0.390	0.155	0.980			3.806
*SLC35G2* expression	0.067	0.244	0.825	0.671	1.014	48.63	65 (7)	Variables not in the multivariate model							
*TMEM61* expression	0.073	0.285	0.733	0.522	1.029	32.23	59 (5)								
Symptoms	0.084	0.303	3.789	0.834	17.211	48.52	66 (7)								

### TMEM expression in PBMCs of ccRCC patients

**Fig. 4:**
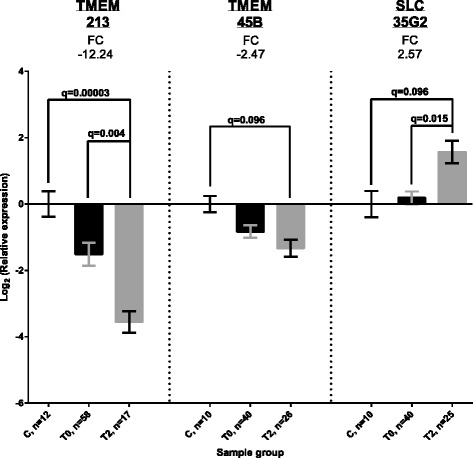
A comparison of TMEM expression in PBMCs. Average log_2_ relative expression data in each sample group ± standard error of mean is shown in each chart. FC – fold-change. C – control samples (PBMCs from healthy volunteers). T0 – PBMCs obtained from patients before nephrectomy. T2 – PBMCs obtained from patients 12 months after surgery. n – number of samples

To assess the utility of TMEM expression as potential biomarkers we analyzed their expression in PBMCs of ccRCC patients (see Table 1). According to the Illumina Human Body Map 2.0 project (NCBI Gene Expression Omnibus accession no. GSE30611) six out of ten selected TMEMs were found to be expressed in PBMCs and those were included in further analysis (i.e., *SLC35G2*, *TMEM30B*, *TMEM45A*, *TMEM45B*, *TMEM116* and *TMEM213*). Although a comparison between expression of TMEMs in PBMCs obtained from patients before nephrectomy (T0) and from healthy volunteers did not reveal statistically significant differences, we observed significant down-regulation of *TMEM213* (fold-change = −12.24, q < 0.0001, *n* = 17), down-regulation of *TMEM45B* (fold-change = −2.47, q < 0.1, *n* = 26) and up-regulation of *SLC35G2* (fold-change = 2.57, q < 0.1, *n* = 25) between PBMCs of healthy volunteers and PBMCs obtained from patients one year post nephrectomy (T2, Fig. 4). Furthermore, we found statistically significant down-regulation of *TMEM213* and up-regulation of *SLC35G2,* as comparing time points before (T0) and 12 months after the surgery (T2), suggesting fluctuation of their expression in PBMCs post-nephrectomy (Fig. 4). No correlation between TMEM expression in tumors and PBMCs was observed (data not shown). Logistic regression did not reveal the dependence of clinical parameters (i.e., tumor size, Fuhrman grade, metastases and progression status) on TMEM expression in PBMCs (data not shown). Univariate Cox regression did not suggest TMEM’s expression to be involved in disease progression or survival.

### Bioinformatic analyses of transmembrane proteins

In order to determine the topology and structure of 10 TMEMs, found deregulated on the mRNA level in ccRCC tumors, extensive bioinformatic structural analysis was performed. Query sequences from NCBI were submitted to Pfam to classify TMEMs into known protein families [44]. Moreover, three independent protein structure prediction gateways: Metaserver [45], PSORT II [47] and Localizome [57] (Table 4) were used to find consensus of the proteins topology with Pfam results.

**Table 4:** Detailed bioinformatic characteristics of TMEMs with topology and function predictions. ^1^ - Metaserver prediction. ^2^ - Localizome prediction, ^3^ - PSORT II prediction. ^4^ - Functional data available; protein localized in Golgi apparatus, endosomes and lysosomes. GI - GenInfo Identifier. TM - transmembrane helix segment. ER - endoplasmic reticulum. Mit – mitochondrial. Nuc – nuclear. Cyt – cytoplasmic. n/a - no result

Property			TMEM213			TMEM207			TMEM61			TMEM45B			TMEM30B			RTP3			TMEM72			TMEM116			SLC35G2			TMEM45A		
Full name			transmembrane protein 213 precursor			transmembrane protein 207 precursor			transmembrane protein 61			transmembrane protein 45B			transmembrane protein 30B			receptor-transporting protein 3			transmembrane protein 72			transmembrane protein 116 isoform 1			solute carrier family 35 member G2			transmembrane protein 45A		
Alternative names			–			UNQ846			–			–			CDC50B			LTM1; TMEM7			KSP37; C10orf127; bA285G1.3			–			TMEM22			DERP7		
NP (NCBI)			001078898.1			997199.1			872338.1			620143.1			001017970.1			113628.1			001116848.1			001180460.1			079522.2			060474.1		
GI			146229352			46409276			32698902			20270331			63003930			13899263			183227675			302058299			148224156			8922243		
Protein isoforms			1			1			1			1			1			1			1			3			1			1		
Transcript variants			1			1			1			1			1			1			1			3			3			1		
Human length (aa)			107			146			210			275			351			232			275			337			412			275		
Pfam			TMEM213 family in eukaryotes; 154 aa; unknown function			n/a			TMEM61 family in eukaryotes; 150–211 aa; unknown function			DUF716 family in metazoa (unknown function) and plants (response to viral attack)			CDC50/ LEM3 (ligand-effect modulator 3) family in glucocorticoid receptor pathway			zf-3CxxC family with zinc-binding domain			n/a			n/a			EamA drug/metabolite transporter-like family with two copies of EamA domain			DUF716 family in metazoa (unknown function) and plants (response to viral attack)		
Tertiary structure prediction^1^			n/a			Similar to TM protein stannin (PDB Id: 1zza)			n/a			n/a			n/a			Similar to Zn-binding proteins (PDB Id: 2cup; 2dkt; 2e2z; 3hcs)			n/a			Similar to human adrenoreceptor (PDB Id: 2rh1; 2r4r) and human adenoreceptor (PDB Id: 3eml)			Similar to TM domain of the multidrug-resistance antiporter from *E. coli* EmrE (PDB Id: 3b5d)			n/a		
TM domains			2	1	1	2	1	1	2	2	2	7	7	7	2	2	2	1	1	1	3	4	1	6	6	3	10	10	8	7	7	5
^1^	^2^	^3^																														
Predicted topology^3^			Type 1a with cyt tail (92 to 107)			Type 1a with cyt tail (71 to 146)			Type 3a			Type 3b			Type 3a			Type Nt with cyt tail (1 to 211)			Type 2 with cyt tail (1 to 94)			Type 3a			Type 3a			Type 3a		
Cleavable N-terminal signal peptide^3^			Yes (1 to 27)			Yes (1 to 29)			No			No			No			No			No			Yes (1 to 50)			No			No		
Cytoplasmic/ Nuclear discrimination^3^			Nuc (55.5)			Cyt (94.1)			Nuc (70.6)			Cyt (94.1)			Cyt (89)			Cyt (89)			Cyt (94.1)			Cyt (94.1)			Cyt (94.1)			Cyt (94.1)		
Subcellular localization^3^			ER (44.4 %)			ER (44.4 %)			Mit (39.1 %) Nuc (34.8 %)			ER (66.7 %)			ER (39.1 %) Mit (39.1 %)			Cyt (30.4 %)			Mit (30.4 %)			ER (43.5 %)			ER^4^ (55.6 %)			ER (77.8 %)		

**Fig. 5:**
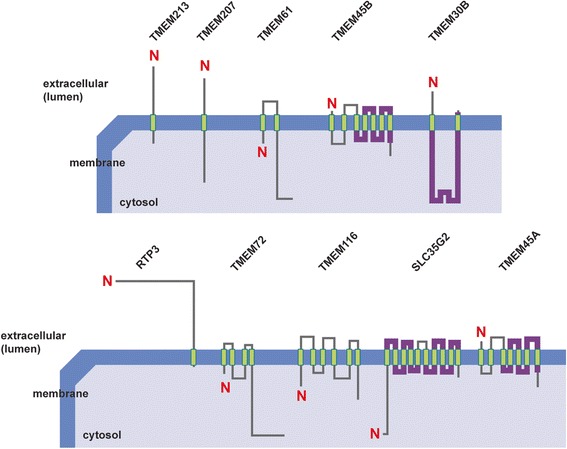
TMEMs - prediction of orientation in plasma membrane. ‘N’ in red indicates N-terminus of proteins, transmembrane segments are in green, functional domains derived from Pfam database are in violet. All structures were generated using Localizome server

TMEM213 was the most down-regulated gene of all TMEMs tested in ccRCC samples. According to Pfam database TMEM213 gene encodes a protein from the family of unknown function and average length of 154 aa, present in all eukaryotes. The Metaserver prediction suggests TMEM213 might contain two transmembrane (TM) helix segments (aa 7–26 and 71–91) with no tertiary homology to any protein crystal structures, currently available in RCSB PDB database. PSORT II classified TMEM213 as type 1a topology protein with one TM domain (aa 75–90). In contrast to Metaserver, PSORT II assigned aa 1–27 as a cleavable N-terminal signal peptide instead of TM domain (Additional file 7: Table S4). PSORT II NNCN Reinhardt’s method for cytoplasmic/nuclear discrimination [52] scored TMEM213 as nuclear protein with reliability of 55.5. k-NN prediction for subcellular localization [56] determined TMEM213 to be connected to endoplasmic reticulum with probability of 44.4 %. Localizome analysis resulted in similar prediction to PSORT II (Fig. 5). Similar analyses were performed for the remaining 9 TMEMs as shown in Table 4.

No Pfam results were obtained for TMEM207, TMEM72 and TMEM116. TMEM61 was assigned as a member of TMEM61 family present in eukaryotes with no known function. TMEMs 45A and 45B were found to be DUF716 (Domain of Unknown Function 716) proteins, present in all metazoa, and predicted to modulate response to viral attack in plants. TMEM30B belongs to CDC50/LEM3 (ligand-effect modulator 3) family. LEM3 affects a downstream step of the glucocorticoid receptor pathway. RTP3 (TMEM7) was predicted to contain pairs of CxxC motifs possibly representing a multiple zinc-binding region, characteristic to zf-3CxxC family. SLC35G2 (TMEM22) on the other hand was recognized as EamA drug/metabolite transporter-like family member with two copies of EamA domain also known as AMAC (acyl-malonyl condensing enzyme) transporter.

In order to confirm known homologs identified by Pfam we analyzed the protein sequences with additional fold-recognition methods available at Metaserver Genesilico gateway. TMEM213, TMEM61, TMEM45B, TMEM30B TMEM72 and TMEM45A showed no homologous crystal structures deposited in RCSB PDB. However comparison, evaluation and ranking by the Pcons5 algorithm [46] at Metaserver denoted similarities of the remaining four TMEMs to known proteins. TMEM207 shared similarity with transmembrane protein stannin (PDB Id: 1ZZA), a small protein involved in inhibition of apoptosis) [59]. TMEM116 was inferred similar to human adrenoreceptor (PDB Id: 2RHL and 2R4R) and human adenoreceptor (PDB Id: 3EML). Similarly to Pfam analysis RTP3 homologs were found in a heterogeneous group of Zn-binding proteins with conserved Zn-binding domain (PDB Id: 2CUP, 2DKT, 2E2Z and 3HCS) and SLC35G2 showed similarities to TM domain of the multidrug-resistance antiporter from *E. coli* EmrE (PDB Id: 3B5days) was found. For RTP3 and SLC35G2 we found confirmation of Metaserver prediction with Pfam results.

Employing Singer’s classification of proteins TMEMs have been sorted according to their predicted topology [50]. Majority of TMEMs are classified as type 3a transmembrane proteins (TMEM61, TMEM30B, TMEM116, SLC35G2 and TMEM45A). TMEM 45B is assigned as type 3b transmembrane protein, whereas TMEM72 – type 2. TMEM213 and TMEM207 were grouped as type 1a with a cleavable signal sequence and one transmembrane segment. RTP3 is a protein with no cleavable signal sequence and one transmembrane segment near C-terminus (type Nt).

Subcellular localization consensus analysis at PSORT II connected TMEM213, TMEM207, TMEM45B, TMEM116, SLC35G2 and TMEM45A with endoplasmic reticulum (with reliability of 44.4 %; 44.4 %; 66.7 %; 43.5 %; 55.6 % and 77.8 %, respectively). RTP3 was found to be cytoplasmic (30.4 %) and TMEM72 – mitochondrial (30.4 %). For TMEM61 and TMEM30B we obtained inconclusive results: mitochondrial/nuclear (39.1 %/34.8 %) and ER/mitochondrial (39.1 %/39.1 %), respectively.

## Discussion

Recently published meta-analysis study of differentially expressed genes in ccRCC led us to examine in detail the expression of ten genes encoding transmembrane proteins (TMEMs) in tumor and PBMC samples derived from ccRCC patients [20]. TMEM protein family is characterized by a presence of putative transmembrane domains, as predicted by *in silico* methods, but limited functional data describing their detailed involvement in malignant transformation is available [29].

Majority of ccRCC deregulated TMEMs were assigned, according to Singer’s classification, as type 3 transmembrane proteins characterized by multiple transmembrane domains in a single polypeptide chain [50]. This group was represented by TMEM61, TMEM30B, TMEM116 and TMEM45B down-regulated in ccRCC tumors and up-regulated TMEM22 (SLC35G2) and TMEM45A. TMEM61 belongs to TMEM61 protein family of unknown function. TMEM30B, predicted member of CDC50/LEM3 family of transcription regulators, facilitates a positive regulation of protein exit from endoplasmic reticulum in a cell [60, 61]. Its’ down-regulation was reported in advanced and recurrent samples of meningioma [11]. Similarly, we found more pronounced down-regulation in less differentiated tumors in our data set. TMEM116 showed tertiary structure similarity to human B2-adrenergic G protein-coupled receptor (GPCR) and human A2A adenosine receptor, found functionally deregulated in other cancer types and associated with tumor invasiveness and evasion of immune system [62–64]. Here we found *TMEM116* expression to be a potential independent predictor of overall survival, as shown by Cox regression, with hazard ratio with each 2-fold increase of *TMEM116* expression equal to 0.554. Cellular localization of the four down-regulated TMEMs was predicted as cytoplasmic, with TMEM116 to be likely of endoplasmic reticulum origin.

**Fig. 6:**
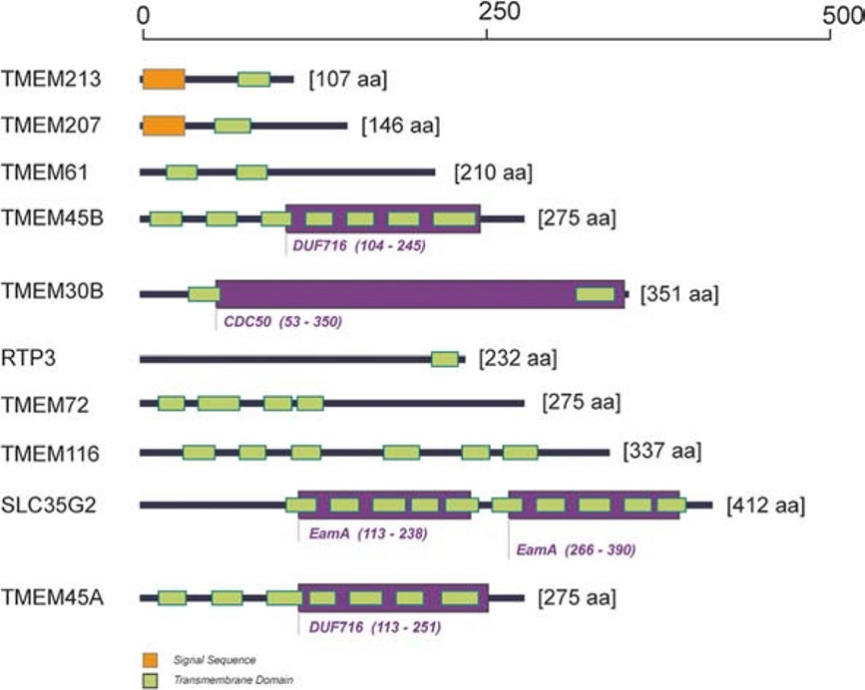
Predicted topologies of TMEMs. Signal sequences are in orange, transmembrane domains in green, functional domains derived from Pfam database in violet. All structures were generated using Localizome server

The up-regulated TMEM22 and TMEM45A encoded proteins were predicted to localize in endoplasmic reticulum. TMEM22 contains a domain similar to E.coli multidrug-resistance antiporter Emre, while TMEM45A (together with TMEM45B) was assigned to DUF716 protein family of unknown function in humans (Fig. 6). TMEM22 (SLC35G2) encodes a member G2 protein of solute carrier family 35, a polytopic transmembrane protein found in Golgi apparatus, endosomes and lysosomes [65, 66] possibly involved in nucleoside-sugar transport [67]. Differential regulation in *SLC35G2* expression in intrahepatic cholangiocarcinoma [68] and promoter hypermethylation of *SLC35G2* in melanoma were previously reported [69]. Dobashi *et al.* found up-regulation of *SLC35G2* in Caki-1 and Caki-2 RCC cell lines and tumor samples; additionally, siRNA silencing of *SLC35G2* diminished cell growth in those cell lines, suggesting its potential involvement in cancer progression and development [29]. Here, we also observe up-regulation of *SLC35G2* expression in our tumor set. Additionally, we find *SLC35G2* expression to be up-regulated in PBMCs collected from patients a year post-nephrectomy, as compared to PBMCs of healthy volunteers. This finding is very promising, although it requires additional conformation in an independent cohort.

TMEM45A is a substrate for ubiquitin ligase and therefore can be degraded in proteasome [70], similarly to TMEM45B, which interacts with ubiquitin-conjugating enzyme E2G 2 [71]. Lee *et al.* observed that progression of ductal carcinoma in situ to invasive breast cancer in xenograft models increased dramatically when *TMEM45A* expression was suppressed [72]. *TMEM45B* down-regulation was detected in squamous lung cell carcinoma, likely due to increased expression of miRNA targeting *TMEM45B* gene [73]. Paulo *et al.* suggests *TMEM45B* regulation by *ERG* and *ETV1* transcription factors in prostate cancer cell lines [74]. *TMEM45B* interacts with tubulointerstitial nephritis antigen (TINAG), which is thought to be involved in FAK/PI3K/Akt-mediated apoptosis [75]. *TINAG* expression is also down-regulated in ccRCC, as shown by microarray experiments [17]. We found that expression decrease of *TMEM45B* may be associated with organ-confined tumors, as shown by *t*-test.

Two proteins: TMEM213 and TMEM207 were assigned to transmembrane protein type 1a with a cleavable signal sequence [50]. Both *TMEM213* and *TMEM207* genes were the most down-regulated in our ccRCC sample set. TMEM213 was predicted to be localized in ER and *in silico* analyses demonstrated existence of TMEM213 protein family with unknown function. TMEM207, with predicted cytoplasmic localization, showed a limited homology to Stannin (SNN), likely involved in mitochondria-mediated apoptosis [76, 77]. Although hierarchical clustering showed that down-regulation of both *TMEM213* and *TMEM207* genes, together with *TMEM72* and *TMEM116* could characterize more advanced ccRCC tumors, these observations require additional confirmation.

TMEM72, classified as a type 2 transmembrane protein, is predicted to be localized in mitochondria, with no similarities to known proteins. We found that strong *TMEM72* down-regulation could be associated with metastases, as revealed by the *t*-test and univariate and multivariate logistic regression. Similar trend is observed in more advanced tumors, as showed by hierarchical clustering.

Previously RTP3 (TMEM7) was classified as type NT transmembrane protein with ER retention signal, is predicted to function in endoplasmic reticulum (ER) membrane [78, 79]. RTP3 showed similarity to zf-3CxxC transcription regulators family, previously reported to be involved in renal cell carcinoma [80, 81]. Zhou *et al.* show that down-regulation or inactivation of *RTP3* was detected in 85 % of primary hepatocellular carcinomas and in 33 % of hepatocellular carcinoma cell lines, likely due to promoter hypermethylation [82]. Although *RTP3* was significantly down-regulated in our tumor set, we did not observe any relevance of this down-regulation in respect to clinical and molecular tumor characteristics.

Interestingly, five of analyzed TMEMs were predicted to have ER membrane localization signals. Endoplasmic reticulum stress has a profound effect on cancer cell proliferation and survival in almost all types of cancer, including RCC [62]. Duivenvoorden *et al.* found that endoplasmic reticulum protein – ERp46 – expression is elevated in ccRCC [83]. Moreover, von Roemeling and colleagues report that inhibition of aberrant stearoyl-CoA desaturase 1 (SCD1) expression attenuates cell proliferation and induces apoptosis in ccRCC cells via the induction of endoplasmic reticulum stress response signaling [84]. Although our findings require further validation, we show that TMEM proteins predicted to be localized in ER (i.e., SLC35G2, TMEM45A, TMEM45B, TMEM116, TMEM207 and TMEM213) can be potentially involved in ccRCC pathogenesis.

## Conclusions

The massive down-regulation of expression of TMEM family members suggests their significant involvement in the pathogenesis of ccRCC. We found that down-regulation of all the TMEMs is most pronounced in advanced tumors and linked their deregulation to metastatic tumors, high Fuhrman grade and disease course. Topology and localization analysis classified majority of the TMEMs as type 3 and type 1 transmembrane proteins, with predicted localization in endoplasmic reticulum, prominently, supporting the involvement of ER proteins in ccRCC pathogenesis.

## Additional files

Additional file 1: Table S2. Clinical characteristics. Detailed clinical characteristics of patients involved in the study at the time of nephrectomy.
Additional file 2: Table S3. Follow-up characteristics. Detailed characteristics of patients’ follow-up. Time to progression – time from nephrectomy to the latest follow-up (in months) or to the follow-up where progression occurred. Time to death – time from nephrectomy to the latest follow-up (in months) or to the death date. In ‘Progression’ and ‘Death’ columns 0 means no progression or death (censored case), 1 means that the disease progressed (type of progression is summarized in ‘Type of progression’ column) or patient died from the disease. N/A – data not available.
Additional file 3: Table S1. Primer sequences used for qPCR TMEM expression measurements.
Additional file 4: Figure S1. TMEM expression in metastatic and non-metastatic tumors. Average log_2_ relative expression data in each sample group ± standard error of mean is shown in each chart. FC – fold-change. *n* – number of samples. M0 – non-metastatic ccRCC. M1 – metastatic ccRCC tissues. q – p-values adjusted for multiple comparisons using Benjamini-Hochberg correction.
Additional file 5: Figure S2. TMEM expression in tumors at early and late stage of the disease, as assessed by TNM staging system. Average log_2_ relative expression data in each sample group ± standard error of mean is shown in each chart. FC – fold-change. *n* – number of samples. pT1/pT2 – organ-confined tumors, as assessed by TNM staging system. pT3/pT4 – advanced tumors, as assessed by TNM staging system. q – p-values adjusted for multiple comparisons using Benjamini-Hochberg correction.
Additional file 6: Figure S3. TMEM expression in tumors comprised of well differentiated or undifferentiated cells, as assessed by Fuhrman grading system. Average log_2_ relative expression data in each sample group ± standard error of mean is shown in each chart. FC – fold-change. *n* – number of samples. G1/G2 – low Fuhrman grade samples. G3/G4 – high Furhman grade samples. q – p-values adjusted for multiple comparisons using Benjamini-Hochberg correction.
Additional file 7: Table S4. Colocalization of topology and function prediction for TMEM213 at Metaserver Genesilico gateway . Colocalization of topology and function prediction for TMEM213 at Metaserver Genesilico gateway. TM helices were superimposed to the results from PSORT II and Localizome.

## Abbreviations

ccRCC: Clear cell renal cell carcinoma
TMEM(s): Transmembrane proteins
ER: Endoplasmic reticulum
qPCR: Quantitative polymerase chain reaction
RTP3: Receptor (chemosensory) transporter protein 3
SLC35G2: Solute carrier family 35, member G2
TMEM30B: Transmembrane protein 30B
TMEM45A: Transmembrane protein 45A
TMEM45B: Transmembrane protein 45B
TMEM61: Transmembrane protein 61
TMEM72: Transmembrane protein 72
TMEM116: Transmembrane protein 116
TMEM207: Transmembrane protein 207
TMEM213: Transmembrane protein 213
RCC: Renal cel carcinoma
VHL: Von Hippel-Lindau syndrome/protein
LOH: Loss of heterozygosity
KNG1: Kininogen 1
MT1A: Metallothionein 1A
PI3K: Phosphatidylinositol-4,5-bisphosphate 3-kinase
AKT: v-Akt murine thymoma viral oncogene homolog
mTOR: Mechanistic target of rapamycin
MTOR: Mechanistic target of rapamycin (serine/threonine kinase)
PTEN: Phosphatase and tensin homolog
PIK3CA: Phosphatidylinositol-4,5-bisphosphate 3-kinase, catalytic subunit alpha
TMEM176: Transmembrane protein 176
TMEM25: Transmembrane protein 25
TMEM127: Transmembrane protein 127
ca.: circa
ANO1: Anoctamin 1, calcium activated chloride channel
TMEM123: Transmembrane protein 123
TMEM165: Transmembrane protein 165
TMEM181: Transmembrane protein 181
TMEM173: Transmembrane protein 173
RT-PCR: Reverse transcription – polymerase chain reaction
RAB37: RAB37, member RAS oncogene family
HIF1A: Hypoxia inducible factor 1, alpha subunit
EPAS1: Endothelial PAS domain protein 1
PBMCs: Peripheral blood mononuclear cells
cDNA: Complementary DNA
CNRQ: Calculated normalized relative quantity
ACTB: beta-actin
ANOVA: one-way analysis of variance
AIC: Akaike Information Criterion
VIF: Variance inflation factor
q-value: p-value corrected by Benjamini-Hochberg method
aa: amino acid
HMM: Hidden Markov model
RSA: Relative solvent accessibility of amino acid
RCSB PDB: Research Collaboratory for Structural Bioinformatics Protein Data Bank
FC: Fold-change
N: number of samples/cases
pT: primary tumor stage
G: Fuhrman grade
r: Pearson correlation coefficient
TNM: Tumor Node Metastases staging system
OR: odds ratio
95 % CI: 95 % confidence interval
ROC AUC: Receiver Operating Characteristics Area Under Curve
HR: Hazard ratio
T0: Time-point before nephrectomy
T2: Time-point one year post-nephrectomy
TM: Trans-membrane
DUF716: Domain of unknown function 716
LEM3: Ligand-effect modulator 3
EamA: O-acetyl-serine/cysteine export gene in *E. coli*
AMAC: Acyl-malonyl condensing enzyme
GPCR: G-protein coupled receptor
ERG: Ets avian erythroblastosis virus E26 oncogene homolog
ETV1: Ets variant 1
FAK: protein tyrosine kinase 2
TMT: Trimethylin
SNN: Stannin
ERK: Mitogen-activated protein kinase 1
ERp46: Thioredoxin domain containing 5
SCD1: Stearoyl-Coenzyme A desaturase 1
