# Supplementary material for: Expression of pre-selected TMEMs with predicted ER localization as potential classifiers of ccRCC tumors
Source: BMC Cancer. 2015 Jul 14;15:518. doi: 10.1186/s12885-015-1530-4 (PMC5015219; doi:10.1186/s12885-015-1530-4)
Supplement: Additional file 1: Table S2. — Clinical characteristics. Detailed clinical characteristics of patients involved in the study at the time of nephrectomy. [file 12885_2015_1530_MOESM1_ESM.docx]

**Table S2. Detailed clinical characteristics of patients involved in the study at the time of nephrectomy.**

| **Sample ID** | **Age** | **Gender** | **Average tumor size [mm]** | **pT** | **pN** | **M** | **Fuhrman grade** |
| --- | --- | --- | --- | --- | --- | --- | --- |
| 1 | 62 | Female | 104 | 4 | 0 | 1 | 4 |
| 2 | 71 | Male | 52.5 | 3 | 0 | 0 | 2 |
| 3 | 66 | Male | 33 | 3 | 0 | 0 | 4 |
| 4 | 77 | Male | 24 | 1 | 0 | 0 | 2 |
| 5 | 74 | Female | 40 | 3 | 0 | 0 | 2 |
| 6 | 52 | Male | 127.5 | 4 | 0 | 1 | 4 |
| 7 | 75 | Male | 21 | 1 | 0 | 0 | 2 |
| 8 | 68 | Female | 44 | 1 | 0 | 0 | 2 |
| 9 | 74 | Female | 42 | 1 | 0 | 0 | 3 |
| 10 | 65 | Male | 45 | 1 | 0 | 0 | 2 |
| 11 | 65 | Male | 67 | 3 | 0 | 0 | 3 |
| 12 | 26 | Male | 40 | 1 | 0 | 0 | 2 |
| 13 | 61 | Male | 43 | 1 | 0 | 0 | 3 |
| 14 | 66 | Male | 75 | 1 | 0 | 0 | 2 |
| 15 | 55 | Male | 64.5 | 1 | 0 | 0 | 3 |
| 16 | 54 | Male | 27 | 1 | 0 | 0 | 2 |
| 17 | 54 | Male | 53.5 | 1 | 0 | 0 | 2 |
| 18 | 62 | Male | 26.5 | 1 | 0 | 0 | 2 |
| 19 | 68 | Female | 26 | 1 | 0 | 0 | 3 |
| 20 | 68 | Male | 65.5 | 3 | 0 | 1 | 4 |
| 21 | 58 | Male | 33 | 1 | 0 | 0 | 2 |
| 22 | 31 | Female | 28.5 | 1 | 0 | 0 | 2 |
| 23 | 62 | Female | 100 | 3 | 0 | 1 | 3 |
| 24 | 53 | Female | 59 | 3 | 0 | 0 | 3 |
| 25 | 80 | Male | 39 | 1 | 0 | 0 | 2 |
| 26 | 63 | Female | 63 | 3 | 0 | 0 | 3 |
| 27 | 67 | Female | 70 | 1 | 0 | 1 | 2 |
| 28 | 56 | Male | 30 | 1 | 0 | 0 | 2 |
| 29 | 78 | Male | 42.5 | 3 | 0 | 0 | 3 |
| 30 | 80 | Female | 38.5 | 1 | 0 | 0 | 1 |
| 31 | 69 | Male | 50 | 3 | 0 | 0 | 2 |
| 32 | 75 | Female | 25 | 3 | 0 | 1 | 2 |
| 33 | 61 | Female | 93 | 3 | 0 | 1 | 2 |
| 34 | 61 | Female | 93 | 3 | 0 | 1 | 2 |
| 35 | 56 | Female | 21 | 1 | 0 | 0 | 2 |
| 36 | 75 | Female | 36 | 1 | 0 | 0 | 2 |
| 37 | 57 | Male | 45 | 1 | 0 | 0 | 4 |
| 38 | 60 | Male | 22 | 1 | 0 | 0 | 1 |
| 39 | 52 | Male | 52 | 1 | 0 | 0 | 2 |
| 40 | 64 | Male | 100 | 3 | 0 | 1 | 3 |
| 41 | 31 | Male | 30 | 1 | 0 | 0 | 2 |
| 42 | 65 | Female | 70 | 3 | 0 | 0 | 3 |
| 43 | 55 | Male | 66 | 3 | 0 | 0 | 3 |
| 44 | 68 | Male | 54.5 | 2 | 0 | 0 | 3 |
| 45 | 60 | Male | 42 | 1 | 0 | 0 | 2 |
| 46 | 65 | Female | 36 | 3 | 0 | 0 | 1 |
| 47 | 53 | Female | 36.5 | 3 | 0 | 0 | 3 |
| 48 | 80 | Female | 70 | 3 | 0 | 0 | 4 |
| 49 | 64 | Female | 35 | 1 | 0 | 0 | 1 |
| 50 | 60 | Female | 59.5 | 3 | 0 | 0 | 4 |
| 51 | 52 | Male | 25.5 | 1 | 0 | 0 | 2 |
| 52 | 69 | Male | 19 | 1 | 0 | 0 | 2 |
| 53 | 59 | Female | 42.5 | 1 | 0 | 0 | 2 |
| 54 | 79 | Male | 71 | 3 | 0 | 0 | 4 |
| 55 | 77 | Female | 72 | 3 | 0 | 1 | 3 |
| 56 | 64 | Male | 41.5 | 3 | 0 | 0 | 4 |
| 57 | 69 | Male | 35 | 3 | 0 | 0 | 2 |
| 58 | 46 | Female | 78 | 1 | 0 | 0 | 3 |
| 59 | 65 | Female | 40 | 1 | 0 | 0 | 2 |
| 60 | 72 | Female | 95 | 3 | 0 | 0 | 3 |
| 61 | 62 | Male | 43 | 1 | 0 | 0 | 2 |
| 62 | 68 | Male | 33 | 3 | 0 | 0 | 3 |
| 63 | 77 | Male | 38 | 3 | 0 | 0 | 2 |
| 64 | 78 | Male | 46 | 1 | 0 | 0 | 3 |
| 65 | 64 | Male | 30 | 1 | 0 | 0 | 3 |
| 66 | 60 | Male | 47.5 | 3 | 0 | 0 | 3 |
| 67 | 57 | Male | 90 | 3 | 1 | 1 | 2 |
| 68 | 53 | Male | 25 | 1 | 0 | 1 | 3 |
| 69 | 47 | Female | 25 | 1 | 0 | 0 | 2 |
| 70 | 72 | Male | 30 | 3 | 0 | 0 | 3 |
| 71 | 84 | Male | 50 | 1 | 0 | 0 | 3 |
| 72 | 74 | Female | 30 | 1 | 2 | 0 | 3 |
| 73 | 59 | Female | 70 | 2 | 0 | 1 | 4 |
| 74 | 52 | Male | 65 | 3 | 1 | 0 | 3 |
| 75 | 73 | Female | 45 | 1 | 0 | 0 | 2 |
| 76 | 73 | Male | 50 | 3 | 0 | 1 | 4 |
