# Supplementary material for: Expression of pre-selected TMEMs with predicted ER localization as potential classifiers of ccRCC tumors
Source: BMC Cancer. 2015 Jul 14;15:518. doi: 10.1186/s12885-015-1530-4 (PMC5015219; doi:10.1186/s12885-015-1530-4)
Supplement: Additional file 2: Table S3. — Follow-up characteristics. Detailed characteristics of patients’ follow-up. Time to progression – time from nephrectomy to the latest follow-up (in months) or to the follow-up where progression occurred. Time to death – time from nephrectomy to the latest follow-up (in months) or to the death date. In ‘Progression’ and ‘Death’ columns 0 means no progression or death (censored case), 1 means that the disease progressed (type of progression is summarized in ‘Type of progression’ column) or patient died from the disease. N/A – data not available. [file 12885_2015_1530_MOESM2_ESM.docx]

**Table S3. Detailed characteristics of patients` follow-up.** Time to progression – time from nephrectomy to the latest follow-up (in months) or to the follow-up where progression occurred. Time to death – time from nephrectomy to the latest follow-up (in months) or to the death date. In ‘Progression’ and ‘Death’ columns 0 means no progression or death (censored case), 1 means that the disease progressed (type of progression is summarized in ‘Type of progression’ column) or patient died from the disease. N/A – data not available.

| **Sample ID** | **Time to progression [months]** | **Time to death [months]** | **Progression** | **Type of progression** | **Death** |
| --- | --- | --- | --- | --- | --- |
| 1 | 8 | 14 | 1 | Metastasis to lungs | 0 |
| 2 | 13 | 48 | 1 | Bladder cancer | 0 |
| 3 | 8 | 8 | 1 | Insulinoma | 0 |
| 4 | 34 | 34 | 1 | Recurrence | 0 |
| 5 | 7 | 7 | 0 | - | 0 |
| 6 | 11 | 11 | 1 | Metastasis to lungs, death from the disease | 1 |
| 7 | 14 | 14 | 0 | - | 0 |
| 8 | 7 | 15 | 1 | Metastasis to bones | 0 |
| 9 | 15 | 15 | 0 | - | 0 |
| 10 | 52 | 52 | 0 | - | 0 |
| 11 | 12 | 12 | 1 | Recurrence | 0 |
| 12 | 14 | 14 | 0 | - | 0 |
| 13 | 51 | 51 | 1 | Tumor in a second kidney | 0 |
| 14 | 51 | 51 | 0 | - | 0 |
| 15 | 50 | 50 | 0 | - | 0 |
| 16 | 34 | 47 | 1 | Papillary thyroid cancer | 0 |
| 17 | 6 | 6 | 0 | - | 0 |
| 18 | 6 | 6 | 0 | - | 0 |
| 19 | 9 | 9 | 0 | - | 0 |
| 20 | 7 | 19 | 1 | Metastasis to lungs | 1 |
| 21 | 49 | 49 | 0 | - | 0 |
| 22 | 6 | 10 | 1 | Ovarian cancer | 0 |
| 23 | 6 | 16 | 1 | Ovarian cancer, death from the disease | 1 |
| 24 | 23 | 23 | 0 | - | 0 |
| 25 | 13 | 13 | 0 | - | 0 |
| 26 | 20 | 20 | 1 | Death from the disease | 1 |
| 27 | 46 | 46 | 0 | - | 0 |
| 28 | 17 | 17 | 0 | - | 0 |
| 29 | N/A | N/A | N/A | - | N/A |
| 30 | 46 | 46 | 0 | - | 0 |
| 31 | 16 | 16 | 0 | - | 0 |
| 32 | 3 | 5 | 1 | Metastasis to lungs, death from the disease | 1 |
| 33 | 23 | 28 | 1 | Metastasis to lungs | 0 |
| 34 | 23 | 28 | 1 | Metastasis to lungs | 0 |
| 35 | 25 | 25 | 0 | - | 0 |
| 36 | N/A | N/A | N/A | - | N/A |
| 37 | 44 | 44 | 0 | - | 0 |
| 38 | N/A | N/A | N/A | - | N/A |
| 39 | 40 | 40 | 0 | - | 0 |
| 40 | 7 | 18 | 1 | Metastasis to lungs, death from the disease | 1 |
| 41 | 19 | 19 | 0 | - | 0 |
| 42 | 42 | 42 | 0 | - | 0 |
| 43 | 39 | 39 | 0 | - | 0 |
| 44 | 19 | 19 | 0 | - | 0 |
| 45 | 7 | 7 | 0 | - | 0 |
| 46 | 41 | 41 | 1 | Urothelial carcinoma of the bladder | 0 |
| 47 | 39 | 39 | 0 | - | 0 |
| 48 | 40 | 40 | 0 | - | 0 |
| 49 | 40 | 40 | 0 | - | 0 |
| 50 | 40 | 40 | 0 | - | 0 |
| 51 | 14 | 24 | 1 | Metastasis to lungs | 0 |
| 52 | N/A | N/A | N/A | - | N/A |
| 53 | 7 | 7 | 0 | - | 0 |
| 54 | 7 | 7 | 0 | - | 0 |
| 55 | 15 | 15 | 1 | Metastasis to brain | 0 |
| 56 | 7 | 7 | 0 | - | 0 |
| 57 | 28 | 28 | 0 | - | 0 |
| 58 | 7 | 7 | 0 | - | 0 |
| 59 | 18 | 18 | 1 | Metastasis to lungs | 0 |
| 60 | 9 | 9 | 0 | - | 0 |
| 61 | N/A | N/A | N/A | - | N/A |
| 62 | 6 | 6 | 0 | - | 0 |
| 63 | 32 | 32 | 1 | Death from the disease | 1 |
| 64 | N/A | N/A | N/A | - | N/A |
| 65 | 9 | 9 | 0 | - | 0 |
| 66 | 6 | 6 | 0 | - | 0 |
| 67 | 7 | 7 | 0 | - | 0 |
| 68 | 4 | 4 | 0 | - | 0 |
| 69 | 14 | 14 | 0 | - | 0 |
| 70 | 6 | 6 | 0 | - | 0 |
| 71 | 11 | 11 | 0 | - | 0 |
| 72 | 11 | 11 | 0 | - | 0 |
| 73 | N/A | N/A | N/A | - | N/A |
| 74 | N/A | N/A | N/A | - | N/A |
| 75 | N/A | N/A | N/A | - | N/A |
| 76 | N/A | N/A | N/A | - | N/A |
