# Supplementary material for: Expression of pre-selected TMEMs with predicted ER localization as potential classifiers of ccRCC tumors
Source: BMC Cancer. 2015 Jul 14;15:518. doi: 10.1186/s12885-015-1530-4 (PMC5015219; doi:10.1186/s12885-015-1530-4)
Supplement: Additional file 3: Table S1. — Primer sequences used for qPCR TMEM expression measurements. [file 12885_2015_1530_MOESM3_ESM.docx]

**Table S1. Primer sequences used for qPCR TMEM expression measurements.**

| **Gene name and primer type** | **Sequence** |
| --- | --- |
| *ACTB* F | 5`-ACAGAGCCTCGCCTTTGCCGAT-3` |
| *ACTB* R | 5`-ATCATCCATGGTGAGCTGGCGG-3` |
| *RTP3* F | 5`-CCTTCGCCAGGTTCCAGT-3` |
| *RTP3* R | 5`-GACTTCTCCTCACTCCAGTTCAT-3` |
| *SLC35G2* F | 5`-AGGTCTTATCTGTGTTAGTTG-3` |
| *SLC35G2* R | 5`-AGAGTCGTAATCTGTATCCA-3` |
| *TMEM30B* F | 5`-GCTGGTCGTCTACATTCGCT-3` |
| *TMEM30B* R | 5`-ATGGCAACTCAGTGTCGTGA-3` |
| *TMEM45A* F | 5`-CAACAACAAAAGCCCAGCCG-3` |
| *TMEM45A* R | 5`-CTCTGAAATTCCCCATGATCCAAC-3` |
| *TMEM45B* F | 5`-GGCACAGGTGTCCTGATGG-3` |
| *TMEM45B* R | 5`-GCGGGTACTTCACTGACCAA-3` |
| *TMEM61* F | 5`-TTCACCACCACACCTTCACC-3` |
| *TMEM61* R | 5`-GCTCCCGTCACACATCTGG-3` |
| *TMEM72* F | 5`-AGCCTCCTACCTACACAAGGGT-3` |
| *TMEM72* R | 5`-CCCACGCCGATCAACACTG-3` |
| *TMEM116* F | 5`-GCCTGATACCTCTGCTATTGA-3` |
| *TMEM116* R | 5`-GCTCGGATAAGTAAGACCATAATG-3` |
| *TMEM207* F | 5`-AACACAGGCACCAAACCACT-3` |
| *TMEM207* R | 5`-CCGAGAGCACCAACTGGAAT-3` |
| *TMEM213* F | 5`-CACTCGGCTTGCTCGGCAGAA-3` |
| *TMEM213* R | 5`-GGCAGAAGTCCACGTTGAGGCA-3` |

F – forward primer

R – reverse primer
