# Supplementary figures and images for: Expression of pre-selected TMEMs with predicted ER localization as potential classifiers of ccRCC tumors
Source: BMC Cancer. 2015 Jul 14;15:518. doi: 10.1186/s12885-015-1530-4 (PMC5015219; doi:10.1186/s12885-015-1530-4)

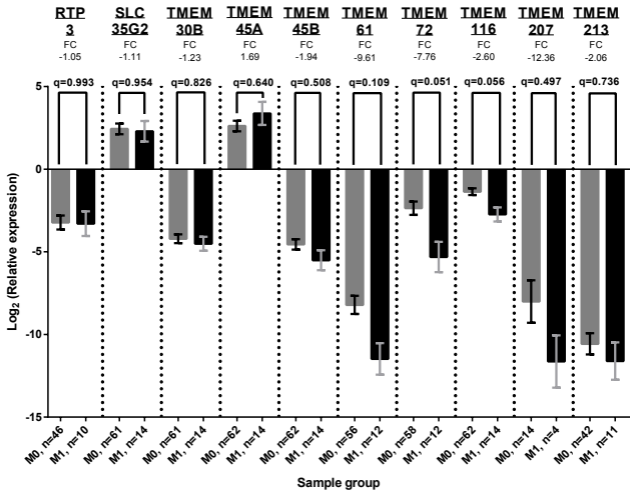

Supplement: Additional file 4: Figure S1. — TMEM expression in metastatic and non-metastatic tumors. Average log2 relative expression data in each sample group ± standard error of mean is shown in each chart. FC – fold-change. n – number of samples. M0 – non-metastatic ccRCC. M1 – metastatic ccRCC tissues. q – p-values adjusted for multiple comparisons using Benjamini-Hochberg correction. [file 12885_2015_1530_MOESM4_ESM.pdf]

| <b>RTP</b> | <b>SLC</b>  | <b>TMEM</b> |
|------------|-------------|-------------|-------------|-------------|-------------|-------------|-------------|-------------|-------------|
| <b>3</b>   | <b>35G2</b> | <b>30B</b>  | <b>45A</b>  | <b>45B</b>  | <b>61</b>   | <b>72</b>   | <b>116</b>  | <b>207</b>  | <b>213</b>  |
| FC         | FC          | FC          | FC          | FC          | FC          | FC          | FC          | FC          | FC          |
| 1.47       | -1.15       | -2.21       | 1.22        | -2.71       | -2.06       | -2.61       | -1.93       | -24.44      | -5.10       |

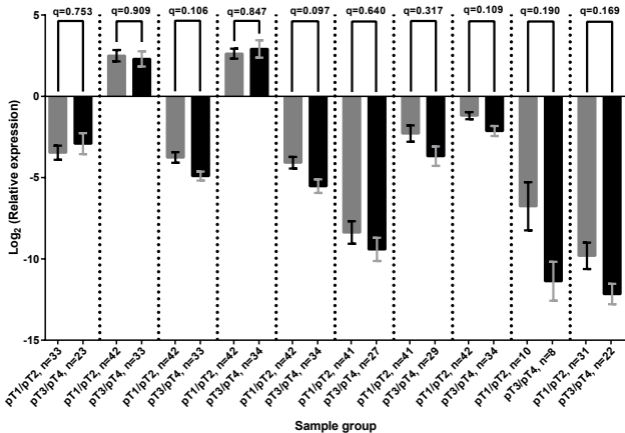

Supplement: Additional file 5: Figure S2. — TMEM expression in tumors at early and late stage of the disease, as assessed by TNM staging system. Average log2 relative expression data in each sample group ± standard error of mean is shown in each chart. FC – fold-change. n – number of samples. pT1/pT2 – organ-confined tumors, as assessed by TNM staging system. pT3/pT4 – advanced tumors, as assessed by TNM staging system. q – p-values adjusted for multiple comparisons using Benjamini-Hochberg correction. [file 12885_2015_1530_MOESM5_ESM.pdf]

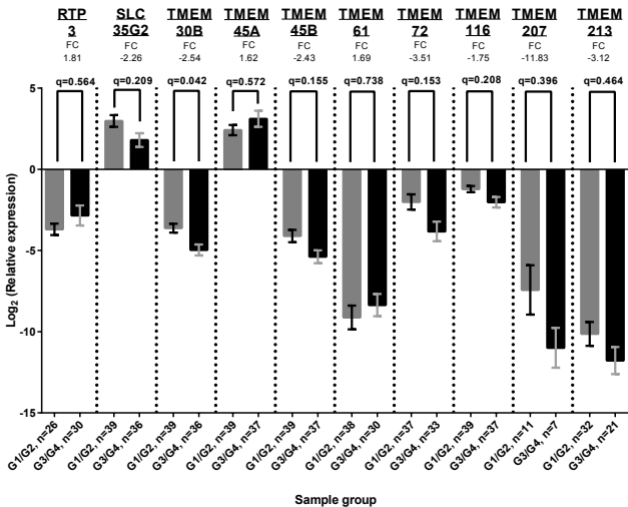

Supplement: Additional file 6: Figure S3. — TMEM expression in tumors comprised of well differentiated or undifferentiated cells, as assessed by Fuhrman grading system. Average log2 relative expression data in each sample group ± standard error of mean is shown in each chart. FC – fold-change. n – number of samples. G1/G2 – low Fuhrman grade samples. G3/G4 – high Furhman grade samples. q – p-values adjusted for multiple comparisons using Benjamini-Hochberg correction. [file 12885_2015_1530_MOESM6_ESM.pdf]
