# Supplementary material for: Expression of pre-selected TMEMs with predicted ER localization as potential classifiers of ccRCC tumors
Source: BMC Cancer. 2015 Jul 14;15:518. doi: 10.1186/s12885-015-1530-4 (PMC5015219; doi:10.1186/s12885-015-1530-4)
Supplement: Additional file 7: Table S4. — Colocalization of topology and function prediction for TMEM213 at Metaserver Genesilico gateway . Colocalization of topology and function prediction for TMEM213 at Metaserver Genesilico gateway. TM helices were superimposed to the results from PSORT II and Localizome. [file 12885_2015_1530_MOESM7_ESM.docx]

**Table S4.** **Colocalization of topology and function prediction for TMEM213 at Metaserver Genesilico gateway.** TM helices were superimposed to the results from PSORT II and Localizome.

| **Parameter** | **Prediction** | | | | |
| --- | --- | --- | --- | --- | --- |
| *Numeration^[[1]](#footnote-1)^* | 1..... | ...10........20..... | ...30........40........50........60........7 | 0........80........90 | ........100..... |
| *Sequence* | MQRLPA | ATRATLILSLAFASLHSACS | AEASSSNSSSLTAHHPDPGTLEQCLNVDFCPQAARCCRTGVDEY | GWIAAAVGWSLWFLTLILLCV | DKLMKLTPDEPKDLQA |
| *TM helices^[[2]](#footnote-2)^*  Metaserver  PSORT II  Localizome | ++++++  SSSSSS  SSSSSS | HHHHHHHHHHHHHHHHHHHH  SSSSSSSSSSSSSSSSSSSS  SSSSSSSSSSSSSSSSSSSS | --------------------------------------------  S-------------------------------------------  SSS----------------------------------------H | HHHHHHHHHHHHHHHHHHHHH  -----HHHHHHHHHHHHHHHH  HHHHHHHHHHHHHHHHHHHHH | ++++++++++++++++  ++++++++++++++++  ++++++++++++++++ |
| *Protein order^[[3]](#footnote-3)^* | DDDDDD | DDDDD-------------DD | DDDDDDDDDDDDDDDDDDDDDD---------------------- | --------------------- | ------DDDDDDDDDD |
| *Protein solvation^[[4]](#footnote-4)^*  NetSurfP_sol25  Soprano_sol25  SPINE_sol25  SPINEX_sol25  ACCpro_sol25  Jnet_sol25  ACCpro_sol5  Jnet_sol5  ACCpro_sol0  Jnet_sol0 | ---B--  ---B--  ---B--  ---BBB  ---B--  B--B--  ---B--  ------  ------  ------ | BBBBBBBBBBBBBBBB--BB  B--BBBBBBBBBBBBBBBBB  B-BBBBBBBBBBBBBBBBBB  BBBBBBBBBBBBBBBBBBBB  BBBBBBBBBBBBBBBBBBBB  B-BBBBBBBBBBBBB-BBB-  B----B--BBB-BB--B-B-  ---BBB-BBBB-B-B-----  --------------------  -------------------- | --B-----------------B--B--B-BB--B--BB---B--B  B-B--B--BBB---B--------B--B-BB--BB-BB--BB--B  --B-------B-B-B-----B--BB-B-BBBBBB-BB-BBB--B  --B----BB-B-B-B-----B--B--B-BB-BBB-BB---BB-B  B-B------BBBBB---------BB-BBBBB-B--BB-BBBBBB  --B------BB---------B--B--B-BB--B--BB--BB---  ----------B-B-------------------B--B----B---  --------------------------------------------  --------------------------------------------  -------------------------------------------- | BBBBBBBBBBBBBBBBBBBBB  BBBBBBBBBBBBBBBBBBBBB  BBBBBBBBBBBBBBBBBBBBB  BBBBBBBBBBBBBBBBBBBBB  BBBBBBBBBBBBBBBBBBBBB  BBBBBBBBBBBBBBBBBBBBB  B-BBBBB-B-B--B--BBBB-  -BBBBBBBBBBBBBBBBBBBB  B--BB----------------  --B---B--BB--BBBBBBB- | --B--B----------  --B--B----------  --BB-B-------B--  --B--B-------B--  B-BB-B----B-----  --B--B-------B--  ----------------  --B--B----------  ----------------  ---------------- |
| *Secondary structure^[[5]](#footnote-5)^* | ------ | --HHHHHHHHHHHHHHHHH- | -------------------------------HHHH--------H | HHHHHHHHHHHHHHHHHHHHH | HHHH------------ |
| *Tertiary structure* | No hits found | | | | |

1. red color indicates superimposition of the parameters to the predicted transmembrane helix segments [↑](#footnote-ref-1)
2. ‘−‘ - outside loop; ‘+’ - inside loop; ‘H’ - transmembrane helix segment; ‘S’ - signaling peptide [↑](#footnote-ref-2)
3. ‘D’ - predicted to be disordered [↑](#footnote-ref-3)
4. ‘B’ - predicted to be buried; ‘−‘ - predicted to be exposed [↑](#footnote-ref-4)
5. ‘H’ - α helix; ‘E’ - β strand [↑](#footnote-ref-5)
